# T Cell/B Cell Collaboration and Autoimmunity: An Intimate Relationship

**DOI:** 10.3389/fimmu.2018.01941

**Published:** 2018-08-27

**Authors:** Lina Petersone, Natalie M. Edner, Vitalijs Ovcinnikovs, Frank Heuts, Ellen M. Ross, Elisavet Ntavli, Chun J. Wang, Lucy S. K. Walker

**Affiliations:** Division of Infection and Immunity, Institute of Immunity and Transplantation, University College London, London, United Kingdom

**Keywords:** follicular helper T cells (Tfh), B cells, germinal center, autoimmunity, costimulation, CD28, CTLA-4, immunotherapy

## Abstract

Co-ordinated interaction between distinct cell types is a hallmark of successful immune function. A striking example of this is the carefully orchestrated cooperation between helper T cells and B cells that occurs during the initiation and fine-tuning of T-cell dependent antibody responses. While these processes have evolved to permit rapid immune defense against infection, it is becoming increasingly clear that such interactions can also underpin the development of autoimmunity. Here we discuss a selection of cellular and molecular pathways that mediate T cell/B cell collaboration and highlight how *in vivo* models and genome wide association studies link them with autoimmune disease. In particular, we emphasize how CTLA-4-mediated regulation of CD28 signaling controls the engagement of secondary costimulatory pathways such as ICOS and OX40, and profoundly influences the capacity of T cells to provide B cell help. While our molecular understanding of the co-operation between T cells and B cells derives from analysis of secondary lymphoid tissues, emerging evidence suggests that subtly different rules may govern the interaction of T and B cells at ectopic sites during autoimmune inflammation. Accordingly, the phenotype of the T cells providing help at these sites includes notable distinctions, despite sharing core features with T cells imparting help in secondary lymphoid tissues. Finally, we highlight the interdependence of T cell and B cell responses and suggest that a significant beneficial impact of B cell depletion in autoimmune settings may be its detrimental effect on T cells engaged in molecular conversation with B cells.

## Introduction

Effective collaboration between T and B cells is a central tenet of protective immunity. Such interactions underlie the development of optimal affinity-matured antibody responses that are required for host defense, permitting the rapid neutralization of bacterial toxins and blockade of viral cell entry. Over the last decade however, it has become apparent that T cell/B cell collaboration also underpins the development of many autoimmune responses leading to undesirable sequelae. Thus, many of the cellular and molecular pathways familiar to us in the context of effective immunity are also implicated in the development of autoimmunity.

In this review we highlight the interdependence of T cell and B cell responses, both in the initiation of humoral immunity and in the context of immune memory. We then home in on the pathways supporting T cell/B cell collaboration and discuss how costimulatory signals orchestrate the chemokine receptor modulation that drives T cell localization to the T-B border and the altered motility that promotes follicular entry. The importance of SLAM family members in stabilizing adhesive interactions between T and B cells is considered, as is the role of cytokines that support or hinder the emergence of T cell help for the B cell response. Next we examine the early work linking follicular helper T cell (Tfh) differentiation to the development of autoimmunity in mice and describe how this prompted a wave of interest in the analysis of blood-borne Tfh-like cells in human autoimmunity. We illustrate how many of the pathways considered earlier are linked to human autoimmunity by probing GWAS datasets for 10 selected autoimmune diseases.

Throughout the review we focus in particular on the Tfh cell subset that enter B cell follicles to support germinal center (GC) formation. However, it is important to note that interactions between B cells and non-Tfh subsets may also play roles in promoting autoimmunity. A recent exciting development in this regard is covered in our final section on T Cell/B Cell Collaboration Outside Secondary Lymphoid Tissues where we discuss the identification of “peripheral helper” T cells that lack bona fide Tfh markers yet appear to provide help to B cells at sites of autoimmune inflammation. Finally, we close the article by discussing the potential to interrupt T cell/B cell collaboration in autoimmune settings by therapeutic B cell depletion.

## Interdependence of T cell and B cell responses

Implicit in the concept of T cell help for B cells is a notion of directionality, implying that T cells are the providers of help and B cells the recipients. However, it has become clear that the reality is far more equitable, with sequential inputs required from both cell types for a successful overall outcome. This is elegantly demonstrated by the molecular underpinnings of the germinal center response, which relies on tightly regulated bi-directional interactions between follicular helper T cells (Tfh) and B cells.

Tfh cell differentiation is a highly complex multistage endeavor [reviewed in (1)], and B cells play an integral role in this process from the moment Tfh cell precursors first interact with B cells at the follicular border in spleen or interfollicular region in lymph nodes (2, 3) and throughout the GC reaction. In the absence of cognate B cells, Tfh precursors expressing Bcl6 (the master transcription factor for Tfh differentiation) fail to assume a mature Tfh cell phenotype within the follicle (3). The maintenance of Tfh cells requires sustained antigenic stimulation and B cells represent the key antigen presenting cell type during the GC reaction (4, 5). Moreover, there is a positive correlation between Tfh cell and GC B cell numbers in GC, emphasizing the intimate functional relationship between the two cell subsets (4, 6).

When it comes to memory responses, T cells play a clear role in the emergence of memory B cells via the GC reaction, and it appears that the inverse is also true, with B cells actively supporting the efficient generation or maintenance of T cell memory (7). Elegant experiments revealed a key role for memory B cells in presenting antigen to memory Tfh cells to drive Bcl6 re-expression (8), and the location of memory Tfh cells in B cell follicles (9, 10) makes them ideally placed for such contacts. In addition to cognate interaction, the role of B cells in T cell memory may include the provision of costimulatory ligands, as well as their contribution to the structural organization and architecture that supports immune responses.

## Pathways supporting T cell/B cell collaboration

Several key pathways regulating T cell/B cell collaboration have been identified over the years (11), and we highlight a number of examples below.

### CD40/CD40L

CD40 and CD40L have long been recognized as key players in humoral immunity and are essential for GC formation (11–13). Blockade of CD40L signaling during an ongoing GC reaction was shown to abrogate the response, emphasizing the need for continuous CD40-CD40L interactions throughout the GC lifespan (14). Clinical studies identified mutations in CD40L as a common cause for human genetic immunodeficiency X-linked hyper-IgM syndrome, where patients presented with impaired GC development emphasizing the importance of T cell/B cell collaboration in the pre-GC stages of adaptive immune responses (11, 15).

### CD28/CTLA-4

**Figure 1:**
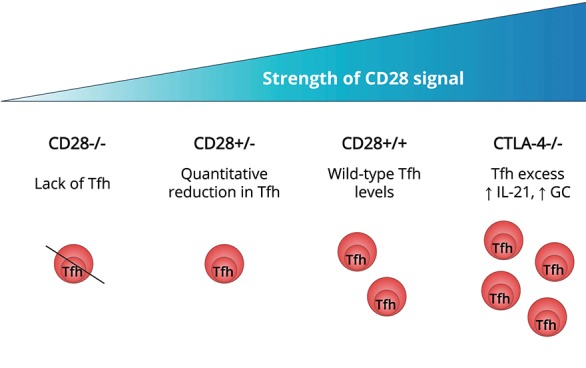
Strength of T cell CD28 engagement influences Tfh differentiation. In mice that are deficient in CD28 signaling, T cells fail to form Tfh (16–18). T cells expressing less CD28, as a result of gene heterozygosity, exhibit a quantitative reduction in Tfh generation compared with wildtype T cells (21). In mice lacking CTLA-4, regulation of CD80 and CD86 is impaired resulting in excessive CD28 engagement. This is associated with spontaneous Tfh induction, increased T cell IL-21 production and formation of germinal centers (21).

Experiments in the late 1990's established that CD28 signaling was required for CD4 T cells to upregulate CXCR5 and migrate into B cell follicles (16), explaining the defect in GC formation in mice lacking CD28 (17) or its ligands (18). CXCR5 induction permits responsiveness to CXCL13 expressed by stromal cells in the follicle and, in association with downregulation of CCR7 (19), guides T cell follicular migration. The G-protein-coupled receptor S1PR2 appears to cooperate with CXCR5 to ensure localization and retention of Tfh at the GC site (20). The amount of CD28 engagement directly influences Tfh differentiation since T cells heterozygous for CD28 showed reduced Tfh induction despite normal activation (Figure 1). The CD28 pathway is regulated by CTLA-4 which binds to the same ligands, CD80 and CD86, but with higher affinity than CD28. Although widely credited with imparting a negative signal, in our view the available evidence does not support this idea and instead suggests that CTLA-4 regulates CD28 engagement by competing for and downregulating their shared ligands (22, 23). The CTLA-4 pathway restricts the formation of Tfh by limiting T cell CD28 engagement (21) and CTLA-4 expression in the regulatory T cell compartment is essential for this process (24, 25). Accordingly, deficiency or blockade of CTLA-4 in mice leads to hyper-engagement of CD28, overproduction of Tfh and spontaneous GC formation (21). CD28 is also required for the development of the follicular regulatory T cells (Tfr) that negatively regulate the GC response (26) (for recent reviews of Tfr please see Wing et al. (27), Fazilleau and Aloulou (28) and Xie and Dent (29) in this collection).

### OX40

The ability of CD28 to promote Tfh development may reflect its capacity to upregulate secondary costimulatory receptors such as OX40 and ICOS. CD28 engagement triggers T cell OX40 upregulation (16) and ligation of OX40 in turn promotes CXCR5 expression (30). Mice expressing OX40L constitutively on dendritic cells showed increased numbers of CD4 T cells in their B cell follicles (31) and conversely deficiency (32) or blockade (33) of OX40 reduced Tfh numbers after viral challenge. Importantly, B cell expression of OX40L has also been shown to support Tfh development (34).

Despite the above, the involvement of OX40 in Tfh differentiation remains controversial; indeed in one study engagement of OX40 was shown to impair Tfh development by promoting expression of Blimp-1 (35) which can inhibit Bcl6 and extinguish the Tfh programme (36). Similarly, in the context of Listeria monocytogenes infection, mice lacking OX40 showed intact Tfh differentiation, and treatment of wildtype mice with agonistic anti-OX40 antibodies expanded effector T cells at the expense of Tfh (37). Thus the involvement of OX40 may be context dependent, with strain-specific and site-specific differences being noted in one study (38). It remains possible that OX40 stimulates the survival or expansion of all differentiated T cells rather than instructing the Tfh differentiation process *per se*.

### ICOS

ICOS is known to be required for the GC response (39–42) and its engagement promotes the differentiation (43) and maintenance (44) of Tfh cells. The level of ICOS upregulation on T cells undergoing activation *in vivo* is tightly coupled to the level of CD28 engagement (21) consistent with the idea that CD28 may promote GC formation via the ICOS pathway. ICOS is superior to CD28 in its capacity to activate phosphoinositide 3-kinase which is known to be required for Tfh cell differentiation and GC formation (6, 45). It has been suggested that ICOS can substitute for CD28 in later phases of the Tfh response (46) although the timing may be critical since extinguishing CD28 at the time of OX40 induction (using OX40-Cre CD28-floxed mice) showed the response was still CD28-dependent at this stage (47, 48). B cells may be an important source of ICOSL since mice lacking B cell-expression of this molecule exhibit significantly reduced Tfh and GC B cell numbers in response to peptide immunization (49, 50). Intriguingly this may reflect a role for ICOSL on bystander (non-cognate) B cells which engages ICOS on T cells approaching the T-B border, promoting their motility and hastening their follicular entry and subsequent Tfh maturation (51). ICOS signaling downregulates the transcription factor Klf2 in both mouse and human T cells and this is critical for ensuring follicular localization of Tfh by keeping CXCR5 high but CCR7, CD62L, PSGL-1, and S1PR1 low (44). Mirroring the findings in murine models, humans with ICOS deficiency show reduced blood Tfh cell frequencies and defects in GC and memory B cell formation (52, 53).

### SLAM family members

During a GC reaction, T and B cells are required to repeatedly engage with each other to facilitate interactions between the receptor/ligand pairs described above. At the T-B border, early interactions between antigen-specific T and B cells are long-lived, while within GC, most cognate Tfh/GC B cell interactions last less than 5 min, but are associated with extensive surface contacts (54, 55). These interactions are stabilized by expression of signal lymphocyte activation molecule (SLAM) family receptors Ly108 and CD84 and SLAM-associated protein (SAP) (56, 57). The importance of these molecules is highlighted by SAP-deficient mice, where Tfh cell differentiation is impaired leading to profound defects in formation of GC, long-lived plasma cells and memory B cells (58–61). Similar observations have been made in X-linked lymphoproliferative disease patients with SAP-deficiency (62).

### Cytokines

IL-2 is a powerful inhibitor of Tfh differentiation (43, 63) by virtue of its STAT5-dependent induction of Blimp-1 (43, 64). Intriguingly, it has been shown that activated dendritic cells in the outer T zone use CD25 expression to quench T cell derived IL-2 thereby generating a microenvironment that favors Tfh formation (65). Tfh differentiation is also influenced by other cytokines, most notably IL-6 in mice (66) and IL-12 in humans (67, 68). Intravital imaging studies have revealed that cognate interactions with GC B cells induce Ca^2+^-dependent co-expression of IL-21 and IL-4 in Tfh (69). These cytokines further promote GC B cell responses, providing a positive feedback loop between Tfh and GC B cells.

## T cell/B cell collaboration in autoimmunity

Widespread recognition of the importance of T cell/B cell collaboration in driving immune-mediated pathology came from a landmark paper in 2009 (70) linking overproduction of Tfh with systemic autoimmunity. This work focused on sanroque mice which have a mutation in the E3 ubiquitin ligase Roquin-1 that regulates mRNA stability and is required for appropriate repression of ICOS expression. Mice with the *Roquin* mutation exhibited high ICOS expression, excessive Tfh formation and lupus-like pathology, however this was abolished if the mice were rendered SAP-deficient, consistent with a critical role for T cell/B cell collaboration in driving this pathology. It was subsequently shown that the *Roquin* mutation dramatically increased progression to type 1 diabetes (T1D) in a TCR transgenic mouse model (71). In a separate mouse model, microarray analysis of T cells responding to pancreatic antigen revealed a striking signature for Tfh differentiation, and cells with a Tfh phenotype showed an enhanced capacity to induce diabetes upon adoptive transfer (72). SAP dependent T cell/B cell interactions have been shown to be essential in the K/BxN model of arthritis (73), where a role for gut microbiota in promoting disease via Tfh induction has been identified (74). A separate study revealed that collagen-induced arthritis could be ameliorated by T cell specific CXCR5 deficiency consistent with the potential involvement of Tfh (75). Findings from mouse models prompted investigation of cells with a Tfh-like phenotype in a wide variety of disease settings in humans, leading to the appreciation that these cells are overrepresented in multiple autoimmune diseases including systemic lupus erythematosus (SLE), Sjögren's syndrome, T1D, myasthenia gravis, rheumatoid arthritis (RA) and multiple sclerosis (MS) (76–78).

The exact provenance of blood-borne cells with a Tfh phenotype has been the subject of much debate. Elegant intravital imaging revealed that while Tfh readily move between GC they only rarely enter the circulation (79). It is widely recognized that Tfh have a circulating memory counterpart (80–83), however expression of many Tfh markers is reduced in the circulation (84, 85) with CXCR5 being least affected (4). Blood-borne CD4+CXCR5+ cells have been shown to be superior at supporting B cell antibody production and class-switching *in vitro* compared to their CD4+CXCR5- counterparts (86–90). Importantly, CXCR5+ cells can be found in the blood of SAP-deficient mice and humans, consistent with the idea that they arise prior to T cell differentiation into mature Tfh within GC (89). Despite their controversial origin and likely heterogeneity, it has become clear that upon antigen exposure circulating Tfh-phenotype cells can migrate to secondary lymphoid tissue and participate in GC reactions suggesting they represent a bona fide functional memory subset (91).

There are many possible explanations for the observed elevation in Tfh-like cells in autoimmune settings. In some cases, this may be secondary to generalized immune activation associated with disease. However, Tfh changes can be detected prior to the onset of overt disease in children at risk of T1D (92), and insulin-specific T cells are enriched for a CXCR5+ Tfh precursor population in children who have only recently developed islet autoantibodies (93). The blood Tfh signature is frequently linked to disease activity (76, 78), and successful treatment of SLE has been shown to decrease Tfh while numbers of Th1 and Th2 cells remain unaltered (94). Persistent antigen has been suggested to favor Tfh differentiation and maintenance (4, 95), so continuous availability of tissue antigen could potentially support this response in chronic autoimmune conditions.

**Figure 2:**
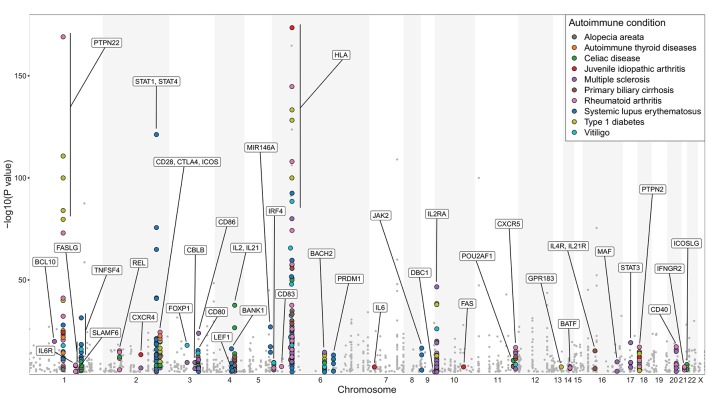
Genes involved in regulating T cell/B cell collaboration are associated with autoimmunity. Manhattan plot of GWAS analysis for 10 selected autoimmune diseases: alopecia areata, autoimmune thyroid diseases (Hashimoto's thyroiditis and Graves' disease), celiac disease, juvenile idiopathic arthritis, MS, primary biliary cirrhosis, RA, SLE, T1D, and vitiligo. Statistical strength of association (-Log_10_*P*) is plotted against genomic position. Single nucleotide polymorphisms (SNPs) in genes with known function in regulating T cell/B cell collaboration are marked with colored circles; other SNPs are marked with gray dots. More than 60% of the highlighted genes are implicated in more than one of the 10 selected autoimmune diseases. The NHGRI-EBI Catalog of published genome-wide association studies (98) was used to generate this figure (catalog version: gwas_catalog_v1.0.2-associations_e92_r2018-05-12; access date 17.05.2018). Only SNP traits with a *p*-value of <1.0 × 10^−5^ in the overall (initial GWAS + replication) population are included in this catalog. For clarity, the y-axis scale used in this figure excludes HLA-DRB1 SNP associations with -Log_10_*P* values of 299, 250 (x2), 185.4 in RA and 224.4, 206, 183 in MS, and an insulin SNP association with -Log_10_P of 185 in T1D. This figure only serves illustrative purposes and no direct comparisons should be made between associations taken from different studies.

The strongest genetic association with autoimmunity maps to the HLA region (96), consistent with its role in presenting the TCR ligands that drive pathogenic and regulatory (97) T cell responses. Interestingly, other genes conferring susceptibility to autoimmunity in humans include many candidates associated with T cell/B cell collaboration. Accordingly, in genome-wide association studies (GWAS) from ten selected autoimmune conditions (T1D, RA, juvenile idiopathic arthritis, autoimmune thyroid diseases, vitiligo, alopecia areata, SLE, MS, primary biliary cirrhosis, celiac disease), polymorphisms in several genes integral for T cell/B cell co-operation bear significant associations with disease susceptibility (Figure 2). These genes are highlighted on the basis of their relevance to T cell/B cell collaboration, however it should be noted that many are also likely to influence T cell interactions with other cell types, such as dendritic cells. A selection of these is discussed below.

### Costimulatory molecules

The *CD28, CTLA4*, and *ICOS* genes are located within a 300 kb region on human chromosome 2 and likely arose from sequential gene duplication (99). Variation at this locus is associated with autoimmunity (100) and blockade of CD28 signaling with CTLA-4-Ig fusion protein is a recognized treatment strategy in a number of autoimmune disease settings (101). As mentioned above, autoimmunity in sanroque mice is associated with de-repression of ICOS mRNA, and dysregulated ICOS expression is also believed to underlie the increase in Tfh and autoimmune phenotype seen in the Sle1 lupus-prone mouse model (102). The genes encoding the ligands for these receptors, *CD80, CD86*, and *ICOSLG*, are also associated with autoimmunity (Figure 2), consistent with the need to tightly control the core pathways that control the induction of T cell help. *CD40*, which provides an essential pathway for GC B cells to perceive T cell help, is implicated in multiple autoimmune diseases (103) as is *DBC1* which regulates its downstream signaling (104). OX40L contributes to pathology in a mouse model of SLE (34) and polymorphisms in *OX40L* (*TNFSF4*) are associated with several diseases where humoral immunity is known to be perturbed including SLE and RA, leading to the investigation of this pathway as a therapeutic target (105).

### Cytokines

Cytokines are important regulators of the GC response, and many of the key cytokines implicated in shaping Tfh and GC B cell differentiation are associated with autoimmune susceptibility. *IL2RA*, which encodes the high affinity subunit for the IL-2-receptor shows one of the strongest associations with T1D outside of the HLA region (106), and as discussed above, IL-2 signaling potently inhibits Tfh differentiation. The *IL2* and *IL21* genes located next to each other on human chromosome 4 (107), and *IL4RA* and *IL21RA* on chromosome 16 (108) also bear a strong association with autoimmunity, potentially reflecting the key roles of IL-21 and IL-4 in orchestrating collaboration between Tfh and B cells within GC (109, 110). Also highlighted by GWAS are *IL6, IL6R*, and *BANK1* which controls IL-6 secretion (111). While IL-12 is considered to be the major cytokine driving Tfh formation in humans (67, 68), this differentiation fate can also be promoted by IL-6. The demonstration that plasmablast-derived IL-6 can promote Tfh differentiation, in a manner that can be inhibited by treatment with the anti-IL-6R antibody tocilizumab (112), highlights a further positive feedback loop between Tfh and B cells.

## Additional genes linked to T cell/B cell collaboration

Other autoimmune-susceptibility genes featured in Figure 2 include the protein tyrosine phosphatase *PTPN22*, which controls the number and activity of Tfh cells (113), and *PTPN2*, deficiency of which leads to increased Tfh cells, GC and autoimmune pathology (114). The chemokine receptors CXCR5 and CXCR4, which play integral roles in regulating cell distribution across GC and facilitating Tfh and GC B cell interactions, are also highlighted (19, 115). The GWAS data also highlight *Gpr183*, the gene encoding the 7α,25-dihydroxycholesterol receptor EBI-2, which must be downregulated for appropriate B cell positioning in GC (116, 117). Indeed forced expression of EBI-2 was shown to diminish the GC response and instead direct B cells to extrafollicular sites (117) while transduction of T cells with an EBI-2 expression vector impaired their capacity to localize to GC (118). Another gene product associated with autoimmunity in this dataset is SLAMF6, which co-operates with SAP to promote T cell/B cell adhesion and is essential for formation of functional GC (119). Importantly, in addition to surface molecules and soluble factors, the GWAS data also draw attention to a number of transcription factors associated with the GC response including BATF, IRF4, Maf, Bob1 (*Pou2af1*), Rel and Blimp-1 (*Prdm1*) (36, 120–125), further highlighting the link between T and B cell interactions and autoimmune susceptibility.

## T cell/B cell collaboration outside secondary lymphoid tissues

Development of tertiary lymphoid structures is frequently seen in chronically inflamed tissues (126), and T cell/B cell collaboration at ectopic sites has been suggested to fuel ongoing autoimmunity (127). Recent findings suggest T cells providing B cell help outside secondary lymphoid organs may bear a distinct phenotype; accordingly Rao et al. described a PD-1^hi^CXCR5^−^CD4^+^ “peripheral helper” T cell population in the synovium of patients with RA which lacked Bcl6 but expressed IL-21, CXCL13, ICOS, and Maf (128). These cells actively promoted memory B cell differentiation into plasma cells *in vitro* and were located adjacent to B cells both inside and outside synovial lymphoid aggregates.

Similarly, in a murine model of airway inflammation, T cells interacting with B cells in the lung exhibited a CXCR5^−^Bcl6^−^ phenotype despite possessing high B cell helper potential, likely *via* their expression of CD40L, IL-21, and IL-4 (129). Remarkably, around 40% of lung-infiltrating B cells in this model showed a GC phenotype implying effective T cell/B cell collaboration, even though the cells were present in loose aggregates rather than well-organized structures.

The relationship of peripheral helper T cells to Tfh cells is currently unclear. However, one study documenting CXCR5^−^BCL6^−^CXCL13^+^ T cells in rheumatoid synovial fluid postulated that these may derive from Tfh cells undergoing progressive differentiation, and loss of CXCR5 and Bcl6 in the synovium (130). The provenance of peripheral helper T cells remains an important question for future clarification.

## Interrupting T cell/B cell collaboration by B cell depletion

Since autoimmunity may arise through over-exuberant T and B cell interactions leading to autoantibody production, depletion of the B cell population has been explored as a treatment strategy. Surprisingly, this has only a moderate effect on serum autoantibody levels, which does not correlate with efficacy, implying an alternative mechanism underlies the beneficial impact (131, 132). Given the interdependence of Tfh and B cell responses highlighted above, one possibility is that B cell depletion affects Tfh cells. Indeed, it has been shown in mice that deletion of GC B cells substantially impairs Tfh homeostasis (4, 133).

In human studies, Xu et al. reported a significant reduction in circulating Tfh frequencies and serum IL-21 levels following B cell depletion with rituximab in patients with T1D, emphasizing Tfh and B cell interdependence in this disease setting (134). Similarly, the elevation in circulating Tfh seen in individuals with Sjögren's syndrome was shown to be normalized by B cell depletion (135).

However, a study by Wallin et al. found no reduction in Tfh numbers in lymph nodes and blood from patients treated with rituximab prior to kidney transplantation (136). This finding may reflect “setting-dependent” roles for B cells in Tfh cell maintenance in humans. Of note, this study identified Tfh cells using CD57 expression which was initially reported to mark GC-resident functionally mature Tfh (137, 138), but was subsequently shown to be expressed by less than a third of GC-resident Tfh cells (139). Therefore, investigating the dynamics of CD57- Tfh cells may be of interest here.

More recently, B cell depletion with ocrelizumab, a humanized anti-CD20 antibody, has been shown to slow disease progression in patients with some forms of MS when compared to placebo or interferon beta-1a treatment (140, 141). Whether B cell depletion impacts Tfh homeostasis in this disease setting is currently unclear, however treatment has been associated with a decrease in cerebrospinal fluid (CSF) T cells as well as B cells and a reduction in CSF levels of the chemokine CXCL13 (142) which can be produced by Tfh cells. Overall, effects on Tfh homeostasis may offer an additional explanation for the efficacy of B cell depletion in certain settings.

## Conclusion

Cooperation between T cells and B cells has been fine-tuned by evolutionary pressures to optimize rapid immune defense. These interactions ensure successful long-term immunity, exemplified by the development of effective T cell and B cell memory. Given that chronic autoimmune diseases may be sustained by the perpetuation, rather than initiation, of self-directed immune responses, the bi-directional interaction between T and B cells may be key to this and may therefore constitute an important therapeutic target.

## Author contributions

LP wrote and edited the manuscript and designed figures. NE designed figures and reviewed and edited the manuscript. VO, FH, ER, EN, and CW reviewed and edited the manuscript. LW conceptualized, wrote and edited the manuscript. All authors approved the final version of the manuscript.

### Conflict of interest statement

The authors declare that the research was conducted in the absence of any commercial or financial relationships that could be construed as a potential conflict of interest.
